# Identification and validation of objective triggers for initiation of resuscitation management of acutely ill non-trauma patients: the INITIATE IRON MAN study

**DOI:** 10.1186/s13049-021-00973-4

**Published:** 2021-11-13

**Authors:** Alexandros Rovas, Efe Paracikoglu, Mark Michael, André Gries, Janina Dziegielewski, Hermann Pavenstädt, Michael Bernhard, Philipp Kümpers

**Affiliations:** 1 Department of Medicine D, Division of General Internal and Emergency Medicine, Nephrology, Hypertension and Rheumatology, University Hospital Münster, Albert-Schweitzer-Campus 1, 48149 Münster, Germany; 2 Emergency Department, University Hospital of Leipzig, Leipzig, Germany; 3 Emergency Department, University Hospital of Düsseldorf, Heinrich-Heine University, Moorenstrasse 5, 40225 Düsseldorf, Germany

**Keywords:** Resuscitation room, Conservative shock room, Nontrauma patients, Emergency department, Trigger factor

## Abstract

**Background:**

While there are clear national resuscitation room admission guidelines for major trauma patients, there are no comparable alarm criteria for critically ill nontrauma (CINT) patients in the emergency department (ED). The aim of this study was to define and validate specific trigger factor cut-offs for identification of CINT patients in need of a structured resuscitation management protocol.

**Methods:**

All CINT patients at a German university hospital ED for whom structured resuscitation management would have been deemed desirable were prospectively enrolled over a 6-week period (derivation cohort, n = 108). The performance of different thresholds and/or combinations of trigger factors immediately available during triage were compared with the National Early Warning Score (NEWS) and Quick Sequential Organ Failure Assessment (qSOFA) score. Identified combinations were then tested in a retrospective sample of consecutive nontrauma patients presenting at the ED during a 4-week period (n = 996), and two large external datasets of CINT patients treated in two German university hospital EDs (validation cohorts 1 [n = 357] and 2 [n = 187]).

**Results:**

The any-of-the-following trigger factor iteration with the best performance in the derivation cohort included: systolic blood pressure < 90 mmHg, oxygen saturation < 90%, and Glasgow Coma Scale score < 15 points. This set of triggers identified > 80% of patients in the derivation cohort and performed better than NEWS and qSOFA scores in the internal validation cohort (sensitivity = 98.5%, specificity = 98.6%). When applied to the external validation cohorts, need for advanced resuscitation measures and hospital mortality (6.7 vs. 28.6%, *p* < 0.0001 and 2.7 vs. 20.0%, *p* < 0.012) were significantly lower in trigger factor-negative patients.

**Conclusion:**

Our simple, any-of-the-following decision rule can serve as an objective trigger for initiating resuscitation room management of CINT patients in the ED.

**Supplementary Information:**

The online version contains supplementary material available at 10.1186/s13049-021-00973-4.

## Background

Trauma patients are regularly admitted directly to the resuscitation room of an emergency department (ED) at dedicated trauma centers. The relevant alert criteria (e.g., trauma mechanism and pattern, Glasgow Coma Scale [GCS] score < 9 points, systolic blood pressure [SBP] < 90 mmHg, need for intubation) are clearly specified by national guidelines [1]. However, there are no comparable triggers for initiating resuscitation room management of critically ill nontrauma (CINT) patients, posing a challenge for their identification by emergency medical services (EMS) or triage nurses.

**Fig. 1:**
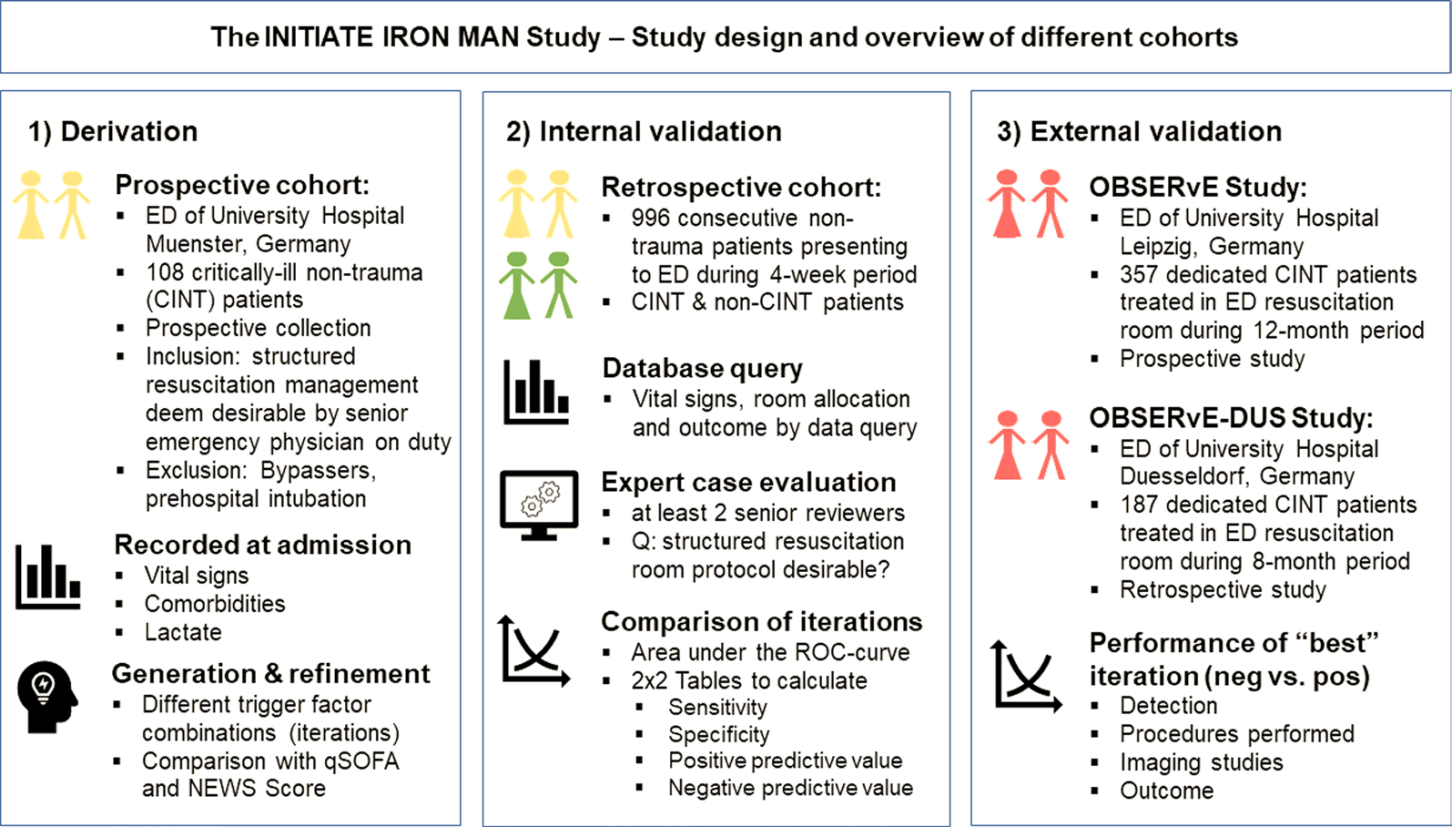
Study design and overview of different cohorts

Early studies on nontrauma resuscitation room management have found that CINT patients account for about 1.5–2.0% of all patient contacts in German EDs [2, 3] and that the nontrauma vs. trauma ratio of resuscitation room patients may be as high as 4:1 in Germany and Belgium [4]. Although the 30-day mortality of CINT patients is around twice that of trauma patients [2], a recent survey showed that only ~ 50% of the participating German EDs use alert criteria to initiate interdisciplinary resuscitation room management of CINT patients, which are mostly limited to classic acute medical conditions triaged according to the Airway, Breathing, Circulation, Disability, and Exposure (ABCDE) scheme [5]. After establishing a structured resuscitation room protocol based on the ABCDE algorithm (Interdisciplinary Resuscitation Room Management of Acutely Ill Nontraumatic Patients [IRON MAN] protocol; Additional file 1: Fig. S1), we recognized that, aside from prehospital intubation [6], there are no objective triggers available in the literature. The aim of this study was therefore to specify and validate objective trigger factor cut-offs for initiating structured resuscitation management in non-intubated CINT patients. A total of 4 different datasets were used: a prospective derivation cohort, a retrospective validation cohort, and two external validation cohorts (Fig. 1).

## Methods

### Study design-overview

The different cohorts in this study are shown in Fig. 1. As a first step, several combinations of quantitative variable thresholds for initiating resuscitation room care were evaluated and optimized in a small prospectively assembled cohort of CINT patients (derivation cohort). The most promising iterations of these trigger factor combinations were compared with established emergency medicine scores. The sensitivity and specificity of the iterations were tested in an independent dataset of consecutive ED patients from the same institution, including non-CINT patients (internal validation cohort). The performance of the most promising trigger factor combination was then evaluated in two large external observational studies (external validation cohorts). The study was carried out in accordance with the Declaration of Helsinki and was approved by the Competent Ethics Committees of the Westphalian-Wilhelms University Münster (no. 2021-017-f-N), University of Leipzig (no. 264-14-25082014), and University of Duesseldorf (no. 2020-960), respectively. The requirement for informed consent was waived.

### Derivation cohort

Over a 6-week period in November and December 2018, all CINT patients admitted to the ED of the University Hospital Muenster were prospectively enrolled (derivation cohort, n = 108). About 16,000 adult nontrauma patients are treated in the ED each year (~ 8000 additional adult trauma patients). The university hospital (highest local emergency care level) and four other municipal hospitals provide emergency care for the city of Münster and the neighboring districts (approx. 1 million inhabitants). CINT patients were defined as patients for whom initiation of a structured resuscitation room management protocol (which was not implemented at that time) would have been deemed desirable *ex post* by the senior ED physician on duty. Typical reasons for this judgment were the need for acute stabilization measures (e.g., initiation of non-invasive ventilation, resuscitation, arterial and central venous line placement, or vasopressor use) and/or need for acute management by several nurses and physicians simultaneously.

Demographic variables, routine biochemistry tests, and physiologic parameters were obtained for each subject during acute care (Table 1). It was irrelevant whether the patients had actually been treated in the resuscitation room or elsewhere in the ED. Emergency patients who bypassed the ER based on predefined treatment pathways, guideline recommendations, or local agreements (e.g., ST-elevation myocardial infarction or prehospital cardiac arrest) were excluded. Prehospital intubated patients were also excluded, as such emergency patients require structured resuscitation management and care anyway. Sepsis was defined according to the second American College of Chest Physicians/Society of Critical Care Medicine (ACCP/SCCM) consensus criteria (Sepsis-2) [7]. The requirement for informed consent for derivation cohorts 1 and 2 and the internal validation cohort (see below) was waived by the Ethics Committee of the General Medical Council Westfalen-Lippe and WWU Münster (no. 2021-017-f-N).

**Table 1:** Clinical characteristics of the study cohorts

Variable	Derivation	Internal validation	External validation	
			*OBSERvE*	*OBSERvE-DUS*
Number of patients, n	108	993	357	187
Female sex	51 (47.2)	478 (48.1)	204 (57.1)	81 (43.4)
Age in years	68 (56–80)	58 (36–72)	73 (59–82)	74 (63–82)
*Reason for admission*				
Sepsis/infection	25 (23.1)	99 (10.0)	16 (4.5)	10 (5.3)
Cardiovascular	19 (17.6)	181 (17.4)	75 (21.0)	48 (25.7)
Neurologic	12 (11.1)	278 (28.1)	79 (21.1)	42 (22.5)
Respiratory	39 (36.1)	68 (6.9)	117 (32.8)	56 (29.9)
Intoxication	9 (8.3)	12 (1.2)	32 (9.0)	9 (4.8)
Hemorrhage	5 (4.6)	32 (3.2)	22 (6.2)	5 (2.7)
Other	9 (8.3)	319 (32.1)	16 (4.5)	17 (9.1)
*Emergency medicine score*				
qSOFA score	1 (1–2)	0 (0–0)	1 (1–2)	1 (1–2)
NEWS score	7 (5–10)	0 (1–2)	Not recorded	Not recorded
*Vital sign*				
SBP	121 (90–144)	139 (125–155)	140 (103–170)	128 (100–164)
SpO_2_ at room air, %	91 (84–97)	98 (97–100)	94 (84–100)	96 (89–98)
RR, breaths/min	21 (16–27)	15 (14–16)	20 (12–35)	20 (16–28)
Admission lactate, mmol/l	2.1 (1.3–3.5)	1.3 (0.9–1.8)	2.5 (1.5–5.6)	2.1 (1.3–3.5)
*Outcome*				
ICU admission	48 (44.4)	65 (6.5)	296 (82.9)	103 (55.1)
In-hospital mortality	17 (15.7)	23 (2.3)	89 (24.9)	29 (15.5)

Data are shown as n (%) or median (interquartile range)

ED, emergency department; ICU, intensive care unit; IQR, interquartile range; qSOFA, Quick Sequential Organ Failure Assessment; SBP, systolic blood pressure; SpO_2_, oxygen saturation

### Internal validation cohort

Electronic chart records from all nontrauma patients admitted to the ED of the University Hospital Muenster in November 2020 (n = 1069) were reviewed by four senior ED physicians with emergency and critical care expertise. Actual room allocation (resuscitation room or elsewhere within the ED) was retrieved from the hospital information system (Orbis; Dedalus Healthcare, Bonn, Germany) by data query. Resuscitation room stays for planned interventions (e.g., cardioversion) were excluded.

After blinding for actual allocation, the reviewers determined for each case whether there had been an indication for a structured resuscitation management (e.g., IRON MAN). Each case was reviewed by at least two referees. If there was no consensus, a third expert (PK) made the final decision. Cases with purely administrative recordings (n = 30; ED bypassing patients) or incomplete/missing data (n = 46; mainly self-presenters) were excluded. The final dataset comprised 993 patients.

### External validation cohorts

#### OBSERvE study

The Observation of Critically Ill Patients in the Resuscitation Room of the Emergency Department (OBSERvE) study [2] was a prospective observational study in 532 CINT patients aged ≥ 18 years treated in the resuscitation room at the ED of the University Hospital of Leipzig (Germany) between September 2014 to August 2015.

#### OBSERvE-Duesseldorf study

The OBSERvE-Duesseldorf (OBSERvE-DUS) study is an ongoing retrospective cohort study of all CINT patients aged ≥ 18 years treated in the resuscitation room of the ED at the University Hospital Duesseldorf. Data on allocation to the resuscitation room from March 2018 to October 2018 were retrieved from the Patient Data Management System (COPRA; COPRA System GmbH, Berlin, Germany) by data query.

In both OBSERvE-cohorts, referral to the resuscitation room was at the discretion of a senior ED physician with expertise in emergency and critical care. The qualitative criteria used for admission to the resuscitation room (no cut-off values were set or specified) are listed in Additional file 3: Table S1 [2]. Numerical trigger factors or thresholds were not specified. After excluding intubated patients (see explanation above) and cases with missing data, the final datasets consisted of 357 patients from the OBSERvE-study and 187 patients from the OBSERvE-DUS study, respectively (Table 1).

### Derivation of objective trigger factor iterations

Unlike other risk or mortality scores, objective trigger factors for initiating resuscitation room management must be readily available upon arrival at the ED and determined before room assignment. We also wanted to capture the enumerative/qualitative ABCDE logic with only a few, objective surrogate parameters. Briefly, we used preliminary decision tree models and receiver-operator characteristic (ROC) curves (SPSS v26, IBM Corporation, Armonk, NY, USA) to identify possible cut-off values for the following (on admission readily available) vital signs: respiratory rate, SBP, and peripheral oxygen saturation (SpO_2_) (A/B); heart rate (C); GCS score (D); and body temperature (E). Subsequently, an interdisciplinary team of intensivists and emergency physicians combined the identified objective cut-offs into several, slightly different trigger factor iterations (hereafter called IRON MAN iterations). These combinations were then tested and further optimized in the derivation cohort (see results for more detail). Frequencies of the vital sign cutoffs that were used are shown in Additional file 2: Fig. S2.

**Fig. 2:**
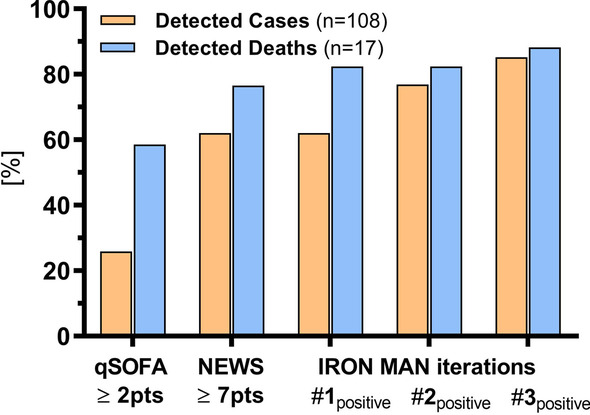
Performance of different trigger factor combinations in the prospective derivation cohort. Bars showing percentage (%) of correctly identified cases (yellow) and detected deaths (blue) depending on a “negative” or “positive” scoring result. ER, emergency room; ICU, intensive care unit; NEWS, National Early Warning Score; qSOFA, Quick Sequential Organ Failure Assessment Score

### Comparison with established severity scores

The IRON MAN iterations were compared with the Quick Sequential Organ Failure Assessment (qSOFA) score [8] and the National Early Warning Score (NEWS) endorsed by the National Health System of the United Kingdom [9]. The NEWS system (0–20 points) recommends “emergency assessment by a clinical team/critical care outreach team with critical-care competencies” at ≥ 7 points; accordingly, a score ≥ 7 was considered as positive (Table 2).

**Table 2:** Components and scores of established scoring systems and most promising trigger factor iterations

	qSOFA (0–3)	NEWS (0–20)	Iteration 1	Iteration 2	Iteration 3
Positive if:	≥ 2 points	≥ 7 points*	≥ 2 points or D alone	qSOFA ≥ 2 or any of the following	Any of the following
A/B	RR > 21/min (1)	RR, breaths per min (0–3): 0, 12–20 1, 9–11 2, 21–24 3, ≥ 25 or < 8 SpO_2_, % (0–3): 0, > 96 1, 94–95 2, 92–93 3, < 91 O_2_ supplement (0–2): No (0) Yes (2)	RR > 25/min or SpO_2_ < 90% (1)	SpO_2_ < 85%	SpO_2_ < 90%
C	SBP ≤ 100 mmHg (1)	HR, beats per min (0–3): 0, 51–90 1, 41–50 or 90–110 2, 111–130 3, 131 SBP, mmHg (0–3): 0, 111–219 1, 101–110 2, 91–100 3, ≥ 220 or ≤ 90	SBP < 100 mmHg (1) Lactate ≥ 3 mmol/l (1)	SBP < 85 mmHg	SBP < 90 mmHg or
D	Altered mentation (1)	AVPU Scale Alert (0), reacting to voice or pain or unresponsive (3)	GCS < 15 (1)	GCS < 15	GCS < 15
E	n.a	Body temperature, °C (0–3): 0, 36.1–38 1, 35.1–36 or 38.1–39 2, > 39.1 3, ≤ 35	n.a	n.a	n.a

AVPU, Alert, Verbal, Pain, Unresponsive; GCS, Glasgow Coma Scale; HR, heart rate; n.a., not available; NEWS, National Early Warning Score; qSOFA, Quick Sequential Organ Failure Assessment Score; RR, respiratory rate; SBP, systolic blood pressure; SpO_2_, peripheral oxygen saturation

*The NEWS score (0–20 points) recommends “emergency assessment by a clinical team/critical care outreach team with critical-care competencies” at ≥7 points. Accordingly, a NEWS ≥7 cut-off was considered as positive

### Outcome measures

In the *derivation cohort*, we analyzed the performance of the scores and different IRON MAN iterations. Outcomes analyzed were:

- the *detection of cases* of (defined as the percentage of cases in the total cohort identified as “positive”), and
- the *detection of deaths* (defined as the percentage of non-survivors (during hospital stay) identified as “positive” by any score or iteration, respectively).

In the *internal validation cohort*, the primary outcome was sensitivity, specificity, and positive and negative predictive values (PPV and NPV), respectively, of the different iterations and comparators. As a secondary outcome, we analyzed whether patients with an indication for a structured resuscitation management had actually been treated in the resuscitation room or elsewhere in the ED.

In the *external validation cohorts*, we compared resource use and 30-day mortality between CINT patients that were “positive” or “negative”, based on the best performing IRON MAN iteration from the internal validation cohort, respectively.

### Statistical analysis

Data are presented as absolute numbers, percentages, or medians with corresponding 25th and 75th percentiles (interquartile range) as appropriate. Differences between groups were analyzed with a two-sided Mann–Whitney U test in the case of continuous variables, and the chi-squared test was used to compare categorical variables. Receiver operator characteristic (ROC) curve analysis was carried out and 2 × 2 contingency tables were used to calculate the area under the ROC curve (AUC), sensitivity, specificity, PPV and NPV for different trigger factor combinations. The distribution of the time-to-event variables were estimated using the Kaplan–Meier method with log-rank testing. All tests were two-sided and statistical significance was set at *p* < 0.05. SPSS v26 (IBM Corporation, Armonk, NY, USA) and Prism v8.4.3 (GraphPad Inc, San Diego, CA, USA) were used for statistical analyses and to plot the data.

## Results

### Derivation and performance of different IRON MAN iterations

The 108 patients in the derivation cohort had a mean age of 68 years and 47% were female. According to the inclusion criteria, none of the patients was intubated by EMS. The most common reason for admission was infection/sepsis, followed by acute cardiovascular, neurologic, or respiratory problems. In total, 44.4% of patients had to be transferred to the ICU, and the in-hospital mortality rate was 15.7% (Table 1).

When looking at individual cases, a key finding was that some patients with “mild” concurrent A/B/C problems could only be identified as positive by *cumulative* iterations. However, patients with e.g., a fulminant *isolated* C problem were more often detected using a conservative any-of-the-following iteration. Another finding was that if the threshold for the GCS score was set to ≤ 13 points, several patients with sole (but critical) D problems would not have been detected at all. To address this problem, we also tried using the cumulative qSOFA score with the original cutoffs (i.e., GCS < 15) in combination very low threshold values for SpO_2_ and SBP (Table 2).

Several approaches were adopted to adequately assess oxygen supplementation; we obtained the best results when we used SpO_2_ under room air (i.e., the value before oxygen administration by EMS) without taking into account the oxygen supplementation that was actually provided. Individual aspects of patient history, comorbidities, or type of admission (i.e., with or without accompaniment by an out-of-hospital emergency physician) had no significant effect on the results (data not shown). The three most promising IRON MAN trigger factor iterations are shown in Table 2.

Overall, iteration 3 showed the highest detection rate (85.2%) in the derivation cohort (Fig. 2). This iteration is an easy-to-remember “any-of-the-following” rule consisting of the following variables (and specific cut-offs): out-of-hospital vasopressor use (mostly Akrinor™, a Cafedrine/Theodrenaline (20:1) Mixture), SBP < 90 mmHg, SpO_2_ < 90%, and GCS < 15 points. Most patients missed by iteration 3 had sole hypotension (SBP range 90–100 mmHg) due to nonpulmonary sepsis or gastrointestinal hemorrhage. Iteration 3 also detected almost all patients who died during hospitalization (88.2%), providing an indirect demonstration of its high specificity (Fig. 2).

### Internal validation

We next tested the internal validity of the three IRON MAN iterations in a consecutive sample of nontrauma patients who presented at our ED in November 2020. According to the blinded chart review, 68 of 993 patients (6.85%) should have been treated by a structured resuscitation management protocol (~ 2.1 per day). The AUC, sensitivity, specificity, PPV, and NPV of the different iterations and comparators are shown in Table 3. Iteration 3 showed the best results, with a sensitivity of 98.5% (i.e., missing just 1 CINT patient) and specificity of 98.6% (i.e., 13 false alarms). Interestingly, the majority of positive patients detected by iteration 3 (93.8%) showed just one abnormal ABCD component (A/B, 17.3%; C, 8.8%; D, 74.6%). Additionally, a combined A/B + D problem was present in only 6.2% of patients.

**Table 3:** Test characteristics of different trigger factors in the internal validation cohort

Trigger factor	AUC (range)	Sensitivity, %	Specificity, %	PPV, %	NPV, %
qSOFA ≥ 2 points	0.56 (0.48–0.64)	11.8	100	100	93.9
NEWS ≥ 7 points	0.66 (0.58–0.74)*	32.4	99.9	95.7	95.3
Iteration 1	0.92 (0.87–0.97)*	85.3	98.7	82.9	98.9
Iteration 2	0.91 (0.86–0.97)*	83.8	98.9	85.1	98.8
Iteration 3	0.99 (0.97–1.00)*	98.5	98.6	83.3	99.9

AUC, area under the receiver operating characteristics curve; NEWS, National Early Warning Score; NPV, negative predictive value; PPV, positive predictive value; qSOFA, Quick Sequential Organ Failure Assessment Score

*p < 0.0001

Most of the 80 patients detected by iteration 3 were assigned to the neurology (52.5%) and internal medicine (42.5%) departments, and the most common Manchester Triage System categories were orange (65.0%) and yellow (27.5%), followed by red and green (both 3.75%). The rate of ICU admissions (32.5 vs. 4.3%; *p* < 0.0001) and deaths (22.5 vs. 0.6%; *p* < 0.0001) was significantly higher among patients identified by iteration 3 compared to those that were not detected by iteration 3.

After unblinding the actual treatment room assignment, we found that 29/68 patients (42.6%) had been originally allocated to normal ED rooms (obvious under-triage). Only 27/925 non-CINT patients (2.9%) treated in a dedicated resuscitation room would not have needed this resource (possible over-triage) based on the experts’ *ex post* opinion. These data are consistent with our clinical impression that CINT patients are often not recognized as such by healthcare professionals when admitted to the ED.

### External validation of iteration 3

**Fig. 3:**
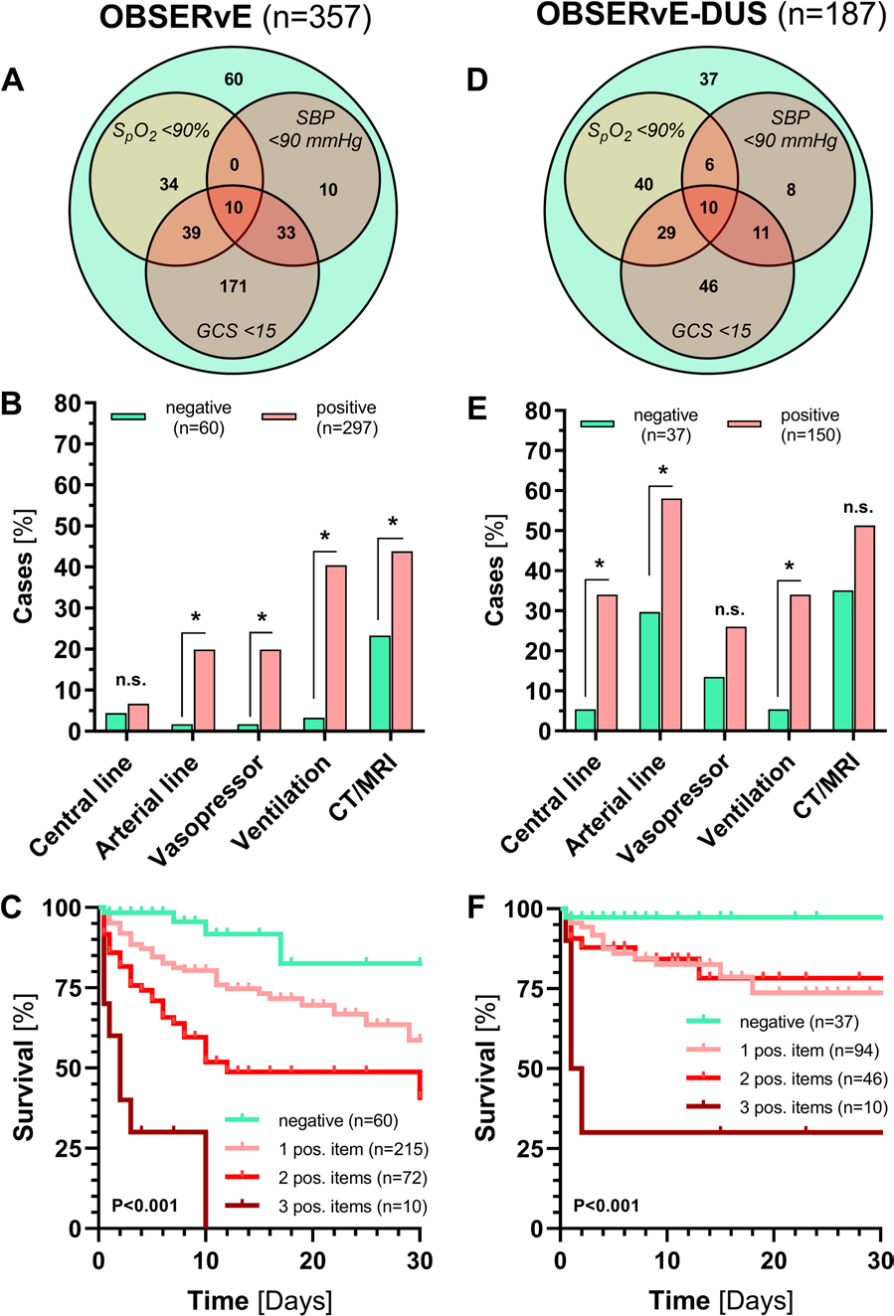
Performance of IRON MAN iteration 3 in the external validation cohorts. **A**, **B** Venn diagram showing the presence or absence of **A**/**B**, **C**, and/or **D** items, respectively. **C**, **D** Bars showing the need for advanced resuscitation measures, such as arterial line placement and mechanical ventilation. **E**, **F** Kaplan–Meier curves of hospital mortality (censored after 30-days) stratified by number of positive items from iteration 3 (Log-rank test)

Comparable datasets are not yet available for external validation. We therefore decided to test IRON MAN iteration 3 in two dedicated resuscitation room cohorts from two other university centers. Of the items in iteration 3, the D item occurred most often alone, but was also frequently found in combination with B or C items (Fig. 3A, D). Iteration 3 detected 297/357 (83.2%) and 150/187 (80.2%) of CINT patients from the OBSERvE and OBSERVE-DUS studies respectively. Compared to correctly detected CINT patients, unrecognized patients (n = 60) in OBSERvE had ST-elevation myocardial infarction (15.0%), cardiac arrythmia (13.3%), hyperdynamic hemorrhagic shock (11.7%), exacerbated COPD (10.0%) or aortic dissection (8.3%). Unrecognized CINT patients in the OBSERvE-DUS study (n = 37) had cardiac arrythmias (35.1%), hypertensive emergencies (24.3%) or stroke (18.9%), respectively. However, the need for advanced resuscitation measures, such as arterial line placement and mechanical ventilation (Fig. 3B, E), as well as observed 30-day mortality was significantly lower in iteration 3-negative compared to iteration 3-positive CINT patients in both the OBSERvE (6.7 vs. 28.6%, *p* < 0.0001) and OBSERvE-DUS (2.7 vs. 20.0%, *p* < 0.012) study (Fig. 3C, F).

## Discussion

In the present INITIATE IRON MAN study, we aimed to identify objective trigger factors for the identification of non-intubated ED patients in need of a structured resuscitation protocol for the first time. Using several complementary patient cohorts, we demonstrated that a relatively simple and pragmatic “any-of-the-following” rule can identify a large proportion of such CINT patients in the ED with high sensitivity and specificity. Specifically, this rule includes as variables out-of-hospital vasopressor use, SBP < 90 mmHg, SpO_2_ < 90%, and GCS score < 15 points. In keeping with the MARVEL-inspired title of the study, the mnemonic for the initiation of the IRON MAN protocol in our ED is therefore “ViSiON rule” (Out-of hospital Vasopressor administration, Systolic Blood Pressure, Oxygen Saturation, Non-normal Consciousness). This rule does not intend to replace diagnosis-based alarm criteria but should be used as an adjunctive vital parameter-based basic default for emergency medical services (EMS) and triage nurses. The added value of this rule is that the initiation of any structured resuscitation protocol is based on specific and entirely objective thresholds—and not on subjective judgment or even gut feeling. However, additional studies are needed to determine whether the ViSiON rule improves patient safety, clinical outcome, and resource utilization in the ED.

In the absence of a true gold standard for the identification of CINT patients in the ED, we compared the performance of ViSiON rule with that of two established severity scoring systems. However, the NEWS was developed to identify patients at risk of early deterioration or death, while the qSOFA provides simple bedside criteria to identify adult non-ICU patients with suspected infection who are likely to have poor outcomes. Thus, a direct comparison between NEWS, qSOFA, and the ViSiON rule was not entirely appropriate, although it did yield the following insights. Firstly, we found that many CINT patients in our EDs apparently have only one main A/B, C, or D problem that cannot be detected with a simple cumulative scoring system such as the qSOFA, despite its more liberal threshold values. Lowering the qSOFA cutoff from ≥ 2 to ≥ 1 is an easy solution to this problem, but this would inevitably increase over-triage (as demonstrated by a decrease in the PPV from 100 to 59.6% in the internal validation cohort; data not shown). Secondly, we were seeking specific variable and cut-off values that are very simple to calculate and readily applicable by EMS and triage personnel. The NEWS in particular is highly discriminatory because of its seven multilevel items; however, as the complex calculation is usually performed manually or with a software application, immediate implementation is not always possible, especially in the case of advance telephone-based notifications by EMS. Additionally, the NEWS provides a cumulative assessment of disease severity rather than detecting individual ABCD problems. Both over-triage and complexity must be minimized in a nontrauma resuscitation room management strategy for CINT patients that is feasible and acceptable. For these reasons, the pragmatic and simple ViSiON rule has potential for successful implementation in the ED.

It should be noted that the derivation of the various trigger factor iterations was carried out in patients from a single university hospital ED and could therefore be biased by local practices and subjective perceptions of the study team. We cannot exclude the possibility that the ViSiON rule may operate differently in EDs at other levels of care, with different sensitivities and specificities. In this context, we must acknowledge that the validation of the IRON MAN iterations in dedicated resuscitation room datasets is not entirely consistent, as they do not represent a true gold standard due to subjective patient allocation. Nevertheless, the detection rate of iteration 3 in the external cohorts (83.2% and 80.2%) was quite comparable to its performance in the derivation cohort (85.2%). This is astonishing, as the vital signs in the studies were only recorded on admission to the resuscitation room, i.e. after they had partially improved or even normalized as a result of the measures taken by the EMS. A/B problems in particular can be masked by out-of-hospital administration of oxygen, making the retrospective score estimation somewhat inaccurate at this point.

Although intubated patients were a priori excluded in the current analysis, the OBSERvE and OBSERvE-DUS study subsets likely represent a negative selection of dedicated shock room patients with very unfavorable prognosis [2, 6]. However, the significantly lower mortality in ViSiON-negative patients shows that a clinically relevant differentiation of CINT patients is still possible in this group. It is tempting to speculate that many of the ViSiON-negative patients were allocated to the resuscitation room based solely on the suspected diagnosis (e.g. stroke, myocardial infarction) and were thus not true CINT patients. This hypothesis is further corroborated by the strikingly decreased need for advanced resuscitation measures in these patients. On the other hand, over-triage by the ViSiON rule in the internal validation cohort was very low (13 false alarms in 993 patients). We are therefore confident, that the ViSiON rule could be a pragmatic and effective tool for patient allocation in the ED.

## Conclusion and outlook

The clear medical need for early recognition and structured care of CINT patients in the ED has emerged in recent years. Objective trigger factors for the initiation of structured resuscitation protocols must be defined to avoid under-triage. Although prospective, multicenter studies are needed for further validation, our findings indicate that the ViSiON rule can facilitate pragmatic identification of ED patients in need of a structured resuscitation protocol based on objective parameters.

## Supplementary Information

**Additional file 1: Fig. S1**. Interdisciplinary Resuscitation Room Management of Acutely Ill Nontraumatic Patients (IRON MAN) protocol. The team generally consists of two doctors (usually an Emergency Physician and an Internist or Neurologist, depending on the case) and at least two nurses. Doctors from other disciplines are alerted as required. The IRON MAN protocol is practiced at our institution at least four times a year in the training center. In addition to lifelike resuscitation manikins, amateur actors are also used.

**Additional file 1: Fig. S2**. Frequencies (%) of vital sign cutoffs used to construct the IRON MAN iterations in the prospective derivation cohorts. Abbreviations: RR, respiratory rate; SBP, systolic blood pressure; SpO_2_, peripheral oxygen saturation; GCS Glasgow Coma Scale.

**Additional file 3: Table S1**. Resuscitation room admission criteria for nontraumatic critically ill patients based on the ABCDE approach in the external validation cohort (OBSERvE study)^†^.

## Data Availability

The datasets used in this study are available from the corresponding author on reasonable request.

## Abbreviations

ABCDE: Airway, breathing, circulation, disability, and exposure
CINT: Critically ill nontrauma (patients)
ED: Emergency department
EMS: Emergency medical services
GCS: Glasgow Coma Scale
ICU: Intensive care unit
IRON MAN: Interdisciplinary Resuscitation Room Management of Acutely Ill Nontraumatic Patients
NEWS: National Early Warning Score
OBSERvE(-DUS): Observation of Critically Ill Patients in the Resuscitation Room of the Emergency Department (-Duesseldorf)
qSOFA: Quick Sequential Organ Failure Assessment
ROC: Receiver operator characteristic
SBP: Systolic blood pressure
SpO_2_: Peripheral oxygen saturation
ViSiOn: Vasopressor, systolic blood pressure, oxygen saturation, non-normal consciousness
