# Supplementary figures and images for: Identification and validation of objective triggers for initiation of resuscitation management of acutely ill non-trauma patients: the INITIATE IRON MAN study
Source: Scand J Trauma Resusc Emerg Med. 2021 Nov 13;29:160. doi: 10.1186/s13049-021-00973-4 (PMC8590263; doi:10.1186/s13049-021-00973-4)

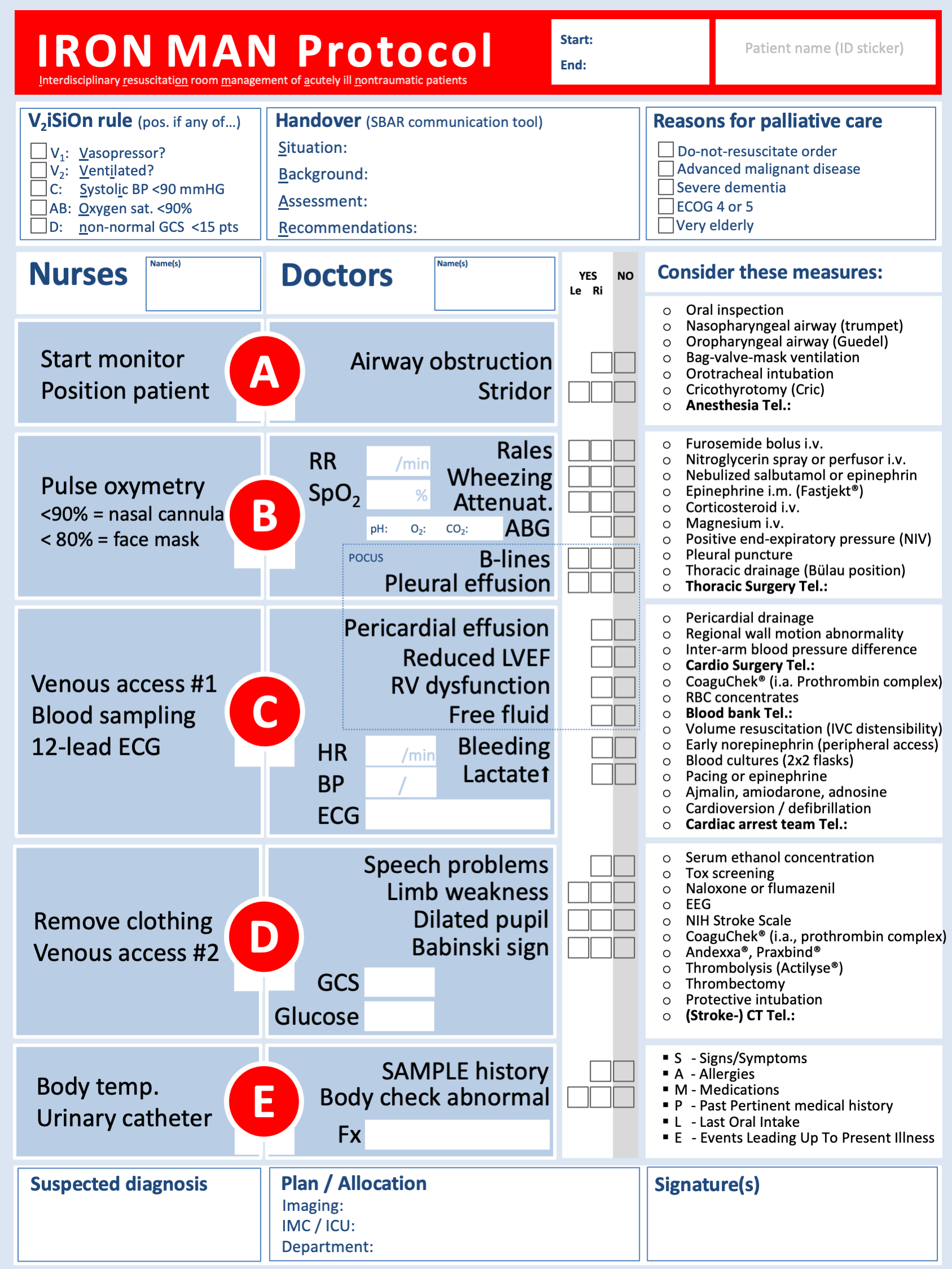

Supplement: Supplementary file 1 — Additional file 1: Fig. S1. Interdisciplinary Resuscitation Room Management of Acutely Ill Nontraumatic Patients (IRON MAN) protocol. The team generally consists of two doctors (usually an Emergency Physician and an Internist or Neurologist, depending on the case) and at least two nurses. Doctors from other disciplines are alerted as required. The IRON MAN protocol is practiced at our institution at least four times a year in the training center. In addition to lifelike resuscitation manikins, amateur actors are also used. [file 13049_2021_973_MOESM1_ESM.tiff]

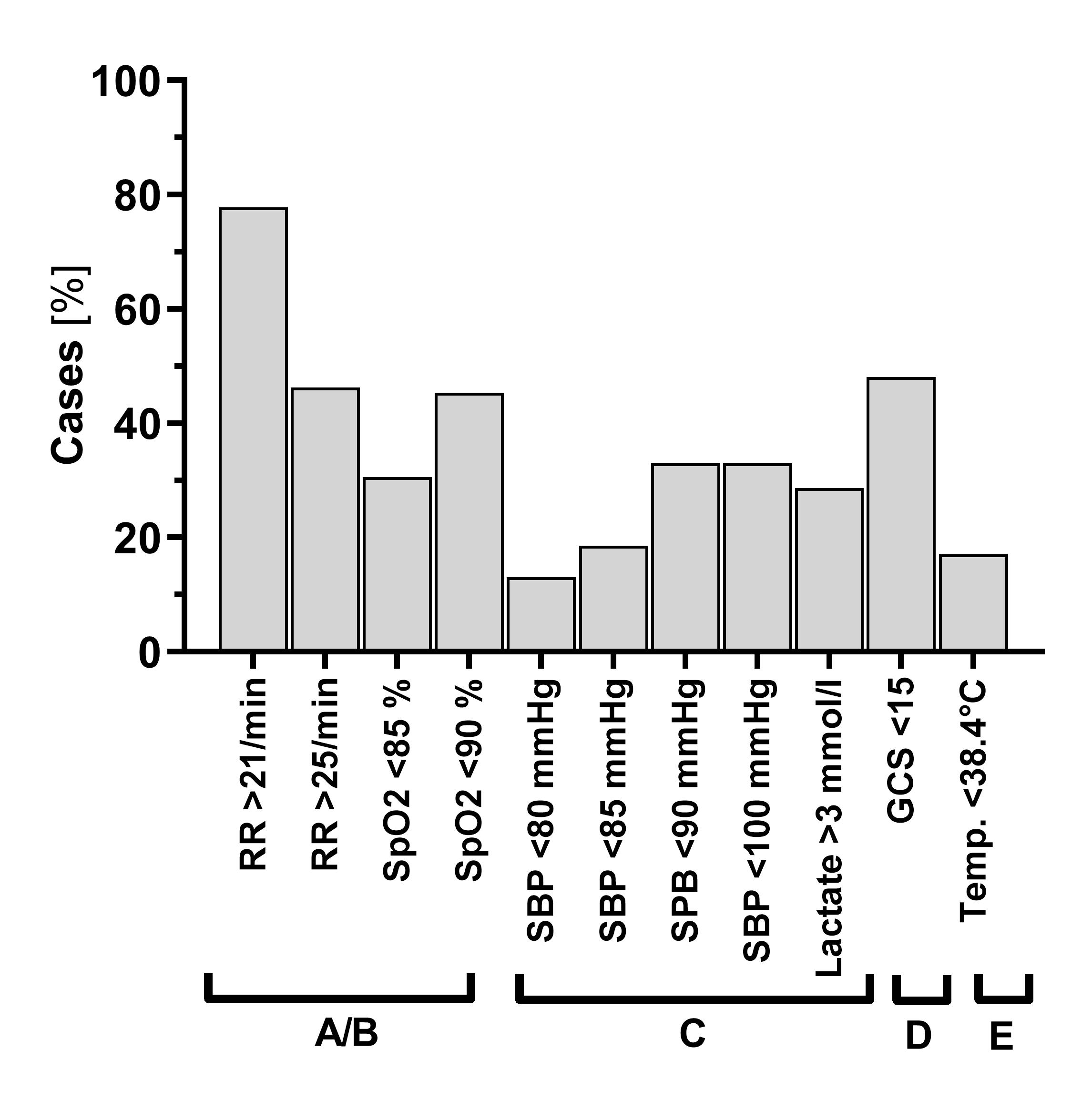

Supplement: Supplementary file 2 — Additional file 1: Fig. S2. Frequencies (%) of vital sign cutoffs used to construct the IRON MAN iterations in the prospective derivation cohorts. Abbreviations: RR, respiratory rate; SBP, systolic blood pressure; SpO2, peripheral oxygen saturation; GCS Glasgow Coma Scale. [file 13049_2021_973_MOESM2_ESM.jpg]
