# Supplementary material for: Identification and validation of objective triggers for initiation of resuscitation management of acutely ill non-trauma patients: the INITIATE IRON MAN study
Source: Scand J Trauma Resusc Emerg Med. 2021 Nov 13;29:160. doi: 10.1186/s13049-021-00973-4 (PMC8590263; doi:10.1186/s13049-021-00973-4)
Supplement: Supplementary file 3 — Additional file 3: Table S1. Resuscitation room admission criteria for nontraumatic critically ill patients based on the ABCDE approach in the external validation cohort (OBSERvE study)†. [file 13049_2021_973_MOESM3_ESM.docx]

**Additional Tables**

**Table S1.** Resuscitation room admission criteria for nontraumatic critically ill patients based on the ABCDE approach in the external validation cohort (OBSERvE study)^†^

| **Problem** | **Example** |
| --- | --- |
| A – Airway | - Enoral swelling (e.g., angioedema, hematoma of the tongue, allergic reaction, abscess) - Free airway at risk - Out-of-hospital airway management (including all alternative devices) |
| B – Breathing | - Respiratory insufficiency with high respiratory rate, rapid deterioration, and low oxygen saturation (oximetry) - Patients requiring rapid airway management |
| C – Circulation | - Circulatory insufficiency (e.g., hypotension, shock of any origin) - Successful or ongoing cardiopulmonary resuscitation - Relevant cardiac arrhythmia (e.g., third-degree AV block, ventricular tachycardia) - Bleeding (e.g., esophageal varices) |
| D – Disability | - Relevant altered mental state - Intracerebral bleeding/stroke |
| E – Environmental | - Intoxication - Hypothermia, hyperthermia - Other critical conditions without ABCD problems |

^†^Senior ED physician in charge activated the resuscitation room depending on individual patient risk.
